# Development of a CD63 Aptamer for Efficient Cancer Immunochemistry and Immunoaffinity-Based Exosome Isolation

**DOI:** 10.3390/molecules25235585

**Published:** 2020-11-27

**Authors:** Zhenguo Song, Jun Mao, Roberto A. Barrero, Peng Wang, Fengqiu Zhang, Tao Wang

**Affiliations:** 1 Department of Pharmacy, Affiliated Cancer Hospital of Zhengzhou University, Zhengzhou 450008, China; zlyysongzhenguo1677@zzu.edu.cn; 2 College of Basic Medical Sciences, Dalian Medical University, Dalian 116044, China; maojun1116@163.com; 3 eResearch Office, Division of Research and Innovation, Queensland University of Technology, Brisbane City QLD 4001, Australia; roberto.barrero@qut.edu.au; 4 College of Nursing and Health, Zhengzhou University, Zhengzhou 450001, China; pengwang@zzu.edu.cn; 5 Henan Key Laboratory of Ion-Beam Bioengineering, Zhengzhou University, Zhengzhou 450000, China

**Keywords:** CD63, aptamer, SELEX, cancer, exosome, breast cancer

## Abstract

CD63, a member of transmembrane-4-superfamily of tetraspanin proteins and a highly N-glycosylated type III lysosomal membrane protein, is known to regulate malignancy of various types of cancers such as melanoma and breast cancer and serves as a potential marker for cancer detection. Recently, its important role as a classic exosome marker was also emphasized. In this work, via using a magnetic bead-based competitive SELEX (systematic evolution of ligands by exponential enrichment) procedure and introducing a 0.5 M NaCl as elution buffer, we identified two DNA aptamers (CD63-1 and CD63-2) with high affinity and specificity to CD63 protein (*K*_d_ = 38.71 nM and 78.43, respectively). Furthermore, CD63-1 was found to be efficient in binding CD63 positive cells, including breast cancer MDA-MB-231 cells and CD63-overexpressed HEK293T cells, with a medium binding affinity (*K*_d_ ~ 100 nM) as assessed by flow cytometry. When immunostaining assay was performed using clinical breast cancer biopsy, the CD63-1 aptamer demonstrated a comparable diagnostic efficacy for CD63 positive breast cancer with commercial antibodies. After developing a magnetic bead-based exosome immunoaffinity separation system using CD63-1 aptamer, it was found that this bead-based system could effectively isolate exosomes from both MDA-MB-231 and HT29 cell culture medium. Importantly, the introduction of the NaCl elution in this work enabled the isolation of native exosomes via a simple 0.5M NaCl incubation step. Based on these results, we firmly believe that the developed aptamers could be useful towards efficient isolation of native state exosomes from clinical samples and various theranostic applications for CD63-positive cancers.

## 1. Introduction

The CD63 protein, the firstly characterized tetraspanin protein encoded on human chromosome 12q13, is a type III lysosomal membrane protein and plays important roles in membrane transport, fusion, and protein kinase signalling [1]. Recently, its role in the metastasis of solid tumors was disclosed [2]. For instance, the high plasma levels of CD63 expression has been regarded as an indicator for chemoresistance in advanced melanoma [3]. In another case, it was found that CD63 could mediate breast cancer malignancy through glycosylation regulation [4]. As a result, CD63 represents a promising target for not only cancer assessment [5,6,7], but also delivering drugs specifically to CD63-positive tumor cells for precision anti-cancer therapy [8].

Although commercial CD63 antibodies are easily accessible, their applications are heavily compromised by factors including the high cost, potential immunogenicity, and instability [9]. The recently developed aptamer technique represents an alternative in this aspect. Also termed chemical antibodies, aptamers are RNA or single-stranded DNA (ssDNA) molecules. In a way similar to antibodies, aptamers display binding capacity to their targets via specific tertiary structure. However, aptamers are developed in vitro via a process called systematic evolution of ligands by exponential enrichment (SELEX) and could be synthesized easily via dry chemistry, which endows aptamers with advantages over their antibody counterparties such as low/no immunogenicity, reduced batch to batch variation, reversible denaturation, efficient tissue penetration (due to smaller size), and accessible to different chemical modifications for enhanced stability and binding affinity [10,11]. Consequently, over the past decades, aptamers targeting different tumor markers such as LDLR [12], EGFR [13], CD133 [14], EpCAM [15,16], PTK7 [17], MUC1 [18], and matrix metalloprotease-9(MMP-9) [19] have been identified and quickly found their way in broad applications ranging from clinical diagnosis, biosensor development, targeted therapy, to drug development [10].

In addition to being a confirmed cancer marker, in recent years, the importance of CD63 in exosome identification has been emphasized [20,21,22,23]. Exosomes are extracellular vesicles with diameter of 50 to 150 nm and featuring a lipid membrane structure [24]. Widely distributed in body fluids, exosomes can be created by virtually all types of cells under both pathological and physiological conditions. Importantly, whether under physiological or pathological conditions, the contents of exosomes are regulated by their cells of origin to play specific functions by transferring information from the parental cells to other cells. In turn, the functional states of particular cells could be assessed by testing the exosome they secreted, which provides the basis for exosome-mediated non-invasive cancer liquid biopsy [20]. Furthermore, exosomes also hold potential in various fields of therapeutics. According to our statistics, there are presently 157 exosome-related clinical trials registered in Clinicaltrials.gov. Indeed, exosome-mediated therapeutics and diagnosis are dramatically changing the landscape of current biological research [8].

For a long time, the issues about how to improve the yield and simplify the procedure of exosome extraction have represented the main limitations that restricted the basic and applied exosome investigations [20]. In recent years, although different methods have been developed, such as ultrafiltration, ultracentrifugation, size-exclusion chromatography, and immunoaffinity, standardized exosome extraction and quantification methods are still not available [20]. For example, although ultracentrifugation has been regarded as the “gold standard” for exosome separation and contributed to most pioneering exosome studies, the expensive equipment, lipoprotein contamination, potential protein aggregate, as well as the damage to the natural structure and functions of the collected exosomes dramatically affect their quantification and functional assessment [25].

It has been observed that certain protein markers that are commonly expressed on exosomes offer a chance for the development of immunoaffinity-mediated exosome separation strategy, via exploring the specific binding between such markers and their affinity agents (e.g., antibodies). Among various exosome markers recognized over the past decades, tetraspanin proteins, especially CD63 and CD81, have been mostly exploited [26], resulting in several antibody-based exosome isolation kits (e.g., Exosome Isolation Kit CD81/CD63 from Miltenyi Biotec and exosome-human CD63 isolation reagent from Thermo Fisher Scientific). A major concern for antibody-mediated exosome separation lies in the difficulty in maintaining the natural structure of the collected exosomes. Even though antibody-based immunoaffinity ensures high-purity exosomes, in addition to the expensive antibody applications, the non-neutral pH and non-physiological elution system (for exosome and antibody separation) used in this strategy detrimentally affects the natural structure and biological functions of the resulted exosomes, which are not favorable for exosome-mediated biomedical study and various therapeutic investigations [27]. Developing aptamers specifically for CD63 marker represents a promising solution to this problem. Apart from low costs, enhanced stability, and high binding affinity, aptamer technique enables the separation of native exosomes with little effort. The binding between aptamers and their targets is strictly decided by their conformational structure, which in turn is determined by different factors, including buffering system and temperature [10]. By modifying the buffer system and ions (e.g., Mg^2+^ and K^2+^) responsible for the specific tertiary structure of aptamers, the binding properties of aptamers can be easily modulated, and result in the release of the captured targets (e.g., exosomes) with intact biological functions.

Over the past five years, a couple of CD63 aptamer-related exosome studies have been reported [28,29,30,31,32,33,34]. However, the current CD63 aptamer related exosome applications focus mainly on high-sensitive exosome detection (via establishing aptamer biosensors) for diagnostic purposes [34,35]; aptamer-based exosome isolation has been rarely investigated. Apparently, developing aptamer-mediated immunoaffinity assay for exosome isolation faces the same problem with the antibody-based exosome isolation. The elution of the separated exosomes from aptamer molecules generally requires a non-natural condition (e.g., heating or low pH incubation), which is unwanted when native exosomes are required. In this work, via introducing a competitive-SELEX procedure, accompanied by using NaCl as an elution system, we endeavor to develop aptamer sequences eligible not only for CD63 positive tumor detection, but also for the development of novel immunoaffinity strategy, enabling the separation of natural state exosomes for various basic and applied applications.

## 2. Results and Discussion

### 2.1. Competitive SELEX Enables the Identification of Aptamers against Native State CD63

**Figure 1:**
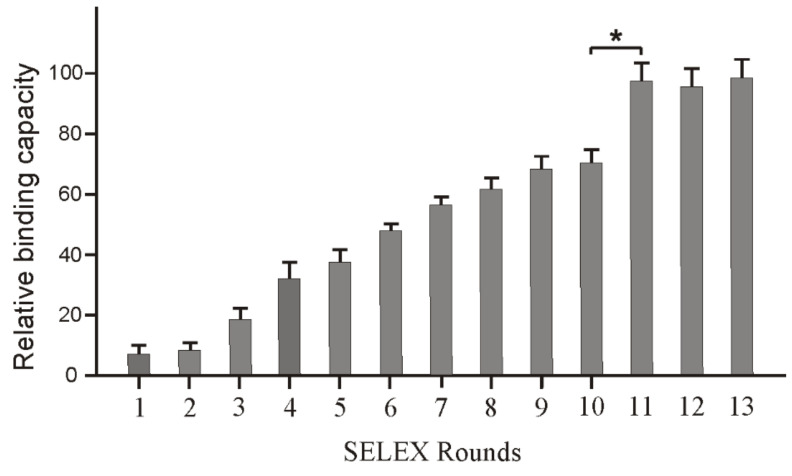
Monitoring the SELEX progress via ELISA assay. Sub-library pools (biotin-labelled) from the indicated SELEX round were co-incubated with CD63 protein in a 96-well nickel plate at room temperature for 30 min. After 1 h of HRP-labelled anti-biotin antibody incubation, the fluorescence intensity was measured by a plate reader (using the QuantaBlu Fluorogenic Peroxidase Substrate). The binding of each round was calculated after subtracting the mean fluorescent intensity of the binding of the original library to CD63 protein. * *p* ≤ 0.05.

Preparation of solid phase protein targets via immobilizing His-tagged protein onto the nickel coated wells (96-well plate) enables simplified SELEX procedure due to easy handling, thorough washing, and quantitative adjustment of the target amount with the progress of SELEX. However, the effectiveness of this target preparation strategy is compromised by two issues. Firstly, CD63 is an extensively glycosylated cell surface protein consisting of four hydrophobic domains. As a result, the tertiary structure of certain domains of the natural protein may undergo substantial changes after the nickel-coated plate-based immobilization, which in the end detrimentally affect the application of the identified aptamers to bind to native human CD63 proteins [22]. Secondly, similarly to other target immobilization methods (e.g., beads-based approach), during target/library incubation, the library sequences might non-specifically bind to the plastic matrices of the wells and result in SELEX failure [6]. In this project, to minimize the chance of enrichment of non-specific binding sequences and to make sure the developed sequences can bind to the native CD63 protein, we intruded a competition-based elution method. As demonstrated in the Material and Method section, following target/library incubation and thorough washing, 500 pmol free CD63 protein dissolved in the incubation buffer (50-fold of the immobilized protein) was loaded into the immobilized CD63/ssDNA complex to competitively elute potential aptamers, which can recognize the free CD63 protein. Through this competitive elution procedure, coupled with a stringent counter selection step, the SELEX process reached a plateau at round 11. As shown in Figure 1, according to our ELISA assay, no significant improvement in binding affinity was recorded from round 11 to 13.

**Figure 2:**
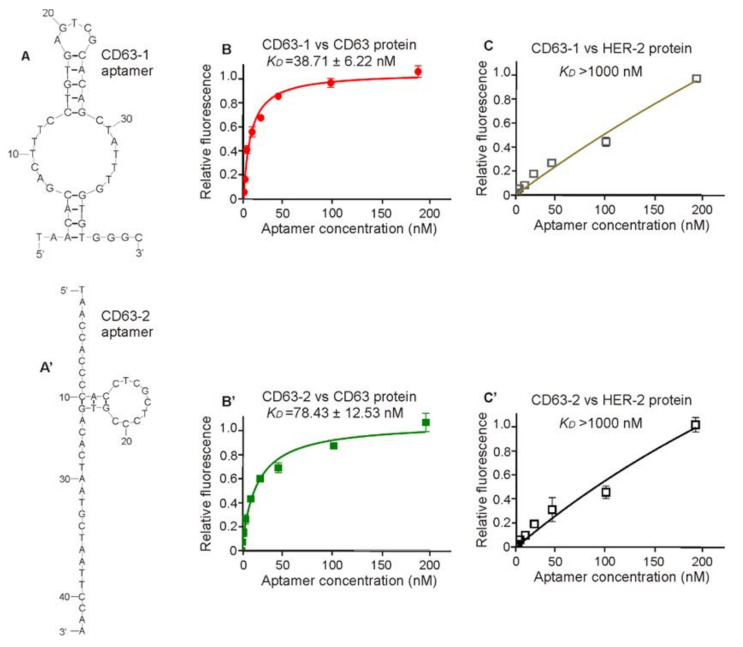
Evaluation of the binding affinity of aptamers to CD63 protein. (**A**,**A’**). The predicted secondary structure of CD63-1 and CD63-2 aptamers using mfold program [36]; (**B**,**B’**). Analysing equilibrium dissociation constants (Kd) of CD63-1 aptamer to CD63 molecule by ELISA; (**C**,**C’**) Testing binding specificity of aptamers to CD63 with HER2 protein as negative control. All data were derived from triplicate tests. Before binding assay, excessive salmon sperm DNAs (10µg/100µL/test) were employed to block the potential binding site of protein targets. Kd was calculated by GraphPad Prism program 3.03.

The competitive-SELEX was then stopped at the 13th round. To enable the identification of potential aptamers from early to last rounds, sub-library pools of round 3, 6, 10, and 13 were submitted to next generation sequencing via a Miseq platform. After data analysis, eight aptamer candidates were selected based on their counts and frequency (sequences with frequency equal or higher than 1%) (Table S1). Due to the potential PCR bias during aptamer development, the sequences showing higher frequency do not necessarily demonstrate higher binding capacity [10]. The binding capacity of the selected eight aptamer candidates were experimentally tested using a fixed 200 nM concentration. As shown in the Supplementary Figure S1, two aptamer candidates, termed CD63-1 (5′-TAACACGACAGACGTTCGGAGGTCGAACCCTGACAGCGTGGGC-3′) and CD63-2 (5′-TAACCACCCCACCTCGCTCCCGTGACACTAATGCTAATTCCAA-3′) (Figure 2A,A’), displaying the best binding property were identified for further assessment.

According to the ELISA assay (Figure 2B,C,B’,C’), both CD63-1 and CD63-2 demonstrated binding capacity to their CD63 protein targets with binding affinity of 38.71 nM and 78.43 nM, respectively. The binding to CD63 protein was specific, as neither CD63-1 nor CD63-2 demonstrated binding to HER2, an irreverent protein. Furthermore, when the binding assay was tested using the starting ssDNA library, no binding to CD63 protein was observed, suggesting the evolution of particular aptamers sequences through the SELEX procedure. Notably, both CD63-1 and CD63-2 sequences did not include the primer binding site (with 43mer). When the whole sequences (including primer binding sites) of CD63-1 and CD63-2 were assessed, the binding capacity of both sequences did not show further improvement, similarly to the previous observation that the constant primer binding sites generally do not contribute to the binding capacity of aptamers [10]. This is likely due to the evolution process of SELEX. During PCR amplification, sequences with the random region displaying strong interaction with the constant primer binding sites may strongly compete for the binding of primer to primer binding sites and therefore show a selective disadvantage for PCR [10]. In contrast, sequences with the random region that do not interact with the constant region are more likely to become dominant over multiple rounds of SELEX. Further truncation to keep the main stem–loop structure of the original aptamers saw complete depletion of the binding captaincy of both aptamers. Importantly, the binding of both aptamers to CD63 protein could be efficiently reversed by a 10 min 0.5 M NaCl incubation (Supplementary Figure S2), which ensured the efficient target dissociation (via NaCl incubation) after aptamer-based immunoprecipitation. As known, for many of the aptamer-based biosensor development (e.g., sandwich lateral flow assay), a pair of aptamers recognize the same target is necessary. Although the exact binding sites of these two aptamers to CD63 molecule are not identified, these two aptamers may provide a basis for the development of high-sensitive sensors for CD63 detection.

### 2.2. CD63-1 Aptamer Is Able to Specifically Recognize CD63 Positive Cells

**Figure 3:**
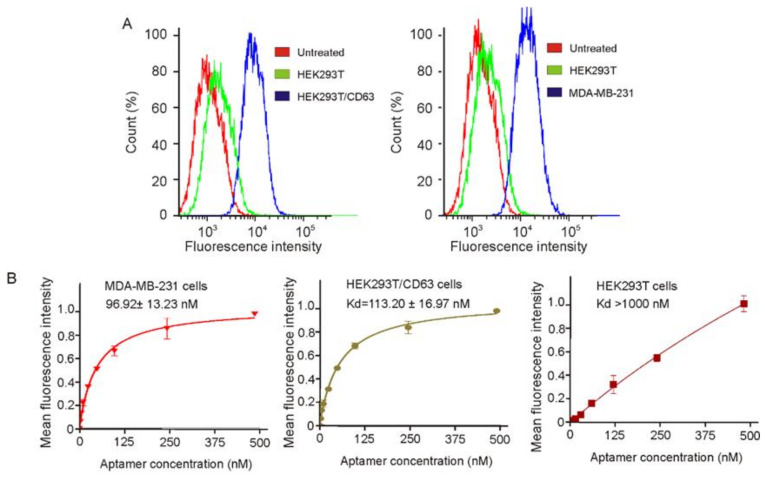
Assessment of binding properties of CD63-1 aptamer to CD63-positive cells. (**A**). FAM-conjugated CD63-1 aptamer was incubated with CD63-positive or -negative cells at 37 °C for 30 min at a concentration of 200 nM, followed by assessing the binding capacity of CD63-1 aptamer to CD63-positive cells with the CD63-negative HEK-293T cells as control; (**B**). Calculating the equilibrium dissociation constants of CD63-1 aptamer to CD63 positive cells via incubating cells with a series of concentrations of aptamer (0–500 nM). Before binding assay, the potential binding motifs of the protein were blocked via using excessive (50µg/500µL/test) salmon sperm DNAs. Kd was calculated via GraphPad Prism program 3.03.

Based on the above results, CD63-1 aptamer, displaying better binding affinity to CD63 protein, was selected for further investigations. For diagnostic and targeted therapeutic applications, the identified aptamer sequences need to bind to CD63 protein molecules in their natural state on the cell surface. To this end, the binding capacity of CD63-1 aptamer to CD63 positive cells was analyzed (Figure 3). Firstly, FAM-tagged CD63-1 aptamer was synthesized and incubated with both CD63-negative HEK293T cells and CD63-positive MDA-MB-231 breast cancer cells at 37 °C for 30 min, followed by flow cytometry assay. For better positive control, a CD63 protein overexpressed HEK293T cell line (HEK293T/CD63) was established (via transient expression of CD63 plasmid to HEK293T cells) and tested with CD63-1 aptamer.

**Figure 4:**
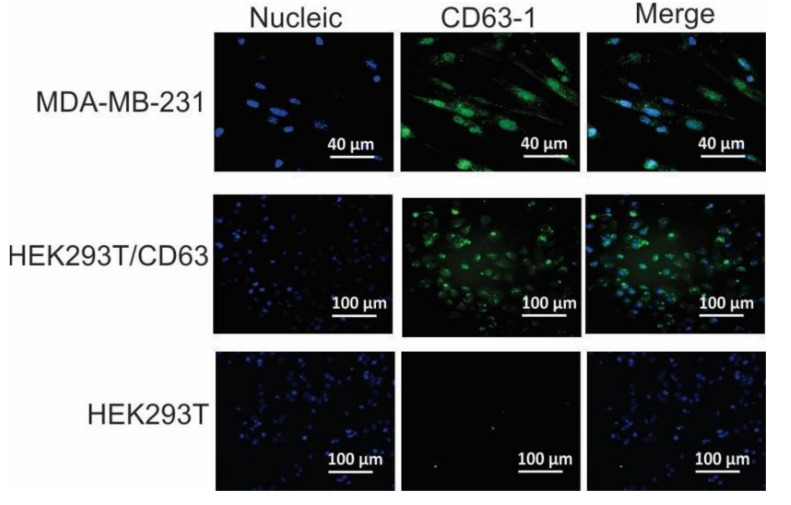
Specific binding of CD63-1 aptamer to CD63-positive cells. After incubating with 200 nM of aptamers for 30 min at 37 °C, cells were imaged using confocal microscopy. Green denotes aptamer (FAM), and blue denotes nuclei (Hoechst 33342). Before performing the test, cells were incubated with excessive (10 µg/500 µL/test) salmon sperm DNA to block the potential binding motif of protein.

As displayed in Figure 3A, differing from the CD63-negative HEK293T cells, both of the tested CD63-positive cell lines showed clear fluorescence signal shift, suggesting that the developed CD63-1 aptamer was able to specifically bind to CD63 positive cells. The subsequent kinetic assays displayed that the CD63-1 aptamer got a medium-high binding affinity to the tested CD63 expressing cells, with dissociation equilibrium constants of 96.92 nM and 113.20 nM for MDA-MB-231 and HEK293T/CD63 cells respectively (Figure 3B). In contrast, no notable fluorescent signal shift and binding property were observed when CD63 negative HEK293T cells were analyzed (Kd > 1000 nM). The specific cellular binding capacity of CD63-1 aptamer was further demonstrated by fluorescence confocal imaging. As displayed (Figure 4), following 30 min incubation using CD63-1 aptamer, while the CD63 negative wild type HEK293T cells did not show aptamer signal, a strong signal was recorded in the CD63 positive MDA-MB-231 breast cancer and HEK293T/CD63 cells, implying the potential of using CD63-1 aptamer for targeted cancer therapeutics.

In fact, the medium-high binding capacity of the CD63-1 displayed to cell surface CD63 protein might present an advantage for targeted therapeutics. In previous clinical trials for EpCAM monoclonal antibody-mediated anticancer therapy, while the high-affinity EpCAM antibodies (Kd from 160 pM to 190 pM) caused acute toxicity in patients [37,38], a medium-high affinity EpCAM antibody with Kd of 91 nM displayed superior safety profiles in patients [39]. Further investigation found that this was because of the ubiquitous expression feature of EpCAM, which is not only expressed with high levels in cancer cells, but also in different normal epithelial cells at low levels [39]. Consequently, although the EpCAM antibody with high binding affinity could efficiently target EpCAM positive cancer cells, it could equally target and affect normal cells with low EpCAM expression. This could be the same for CD63. Even though CD63 protein is highly expressed on certain types of solid cancer cells, it is also displayed in various types of normal cells and is important for membrane transport, fusion process, and protein kinase signaling. For this reason, the developed CD63-1 aptamer, with a moderate binding affinity of around 100 nM to CD63 positive cells, may selectively target CD63 protein overexpressed cancer cells rather than normal tissues.

### 2.3. CD63-1 Aptamer Held Potential for Cancer Detection

**Figure 5:**
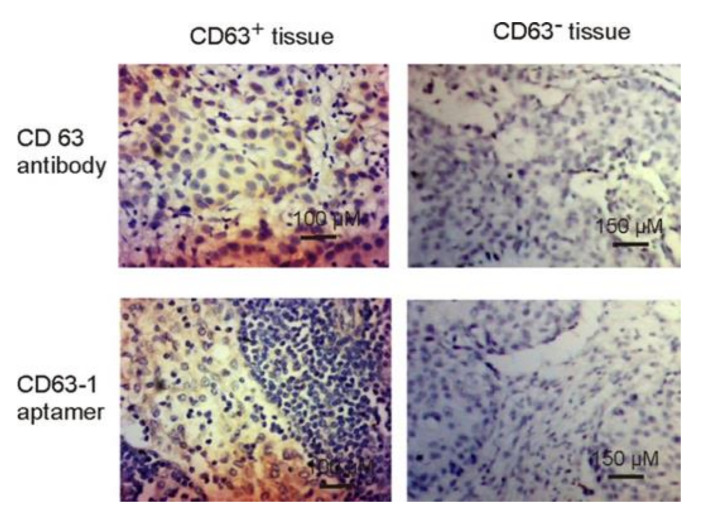
Immunostaining assay of CD63-positive and negative breast cancer tissue. Both CD63 aptamer and CD63 antibody were used. H&E stain was conducted for morphological confirmation.

Although antibody-mediated immunostaining assay is of great importance for current cancer detection [40], the application of antibodies is affected by various factors such as low stability, batch-to-batch variation, and higher costs [41]. Differing from antibodies, aptamers are characterized by enhanced stability (long shelf life), reduced production time and cost, low batch-to-batch variation, and easily accessible to different chemical modifications for improved binding capacity [11,14]. As a result, aptamers hold great promise in immunohistochemistry. In the current project, using clinical breast cancer samples, the potential application of the selected CD63-1 aptamer in CD63 positive cancer diagnosis was assessed. As can be seen from Figure 5, akin to the CD63 antibody, CD63-1 aptamer efficiently bound to cancer cells of the CD63-positive breast cancer sections, while no prominent binding was observed in both CD63-negative breast cancer sections and other types of cells of tumor sections. These observation suggest that CD63-1 aptamer may serve as an agent for CD63 detection in cancer biopsies. Even though only paraffin-embedded breast sections were studied in this work, given the effective recognition of CD63-1 aptamer to CD63 proteins on cell culture (Figure 4 and Figure 5), this aptamer may also be used in other cancer types for immunostaining-based diagnosis.

### 2.4. CD63-1 Aptamer Can Be Used to for the Development of Bead-Based Exosome Isolation System

**Figure 6:**
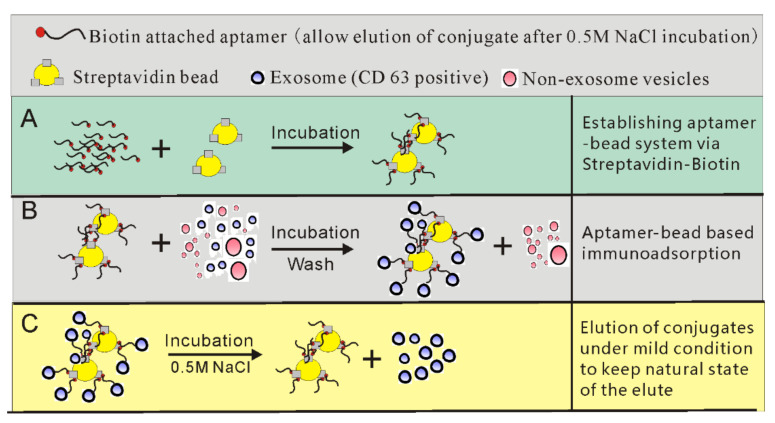
A schematic of the establishment of a bead/aptamer system for native exosome isolation. (**A**) Synthesizing 3′ biotinylated CD63-1 aptamer and incubating with streptavidin magnetic beads to prepare the aptamer–magnetic beads system. (**B**) The aptamer–magnetic beads are incubated with exosome-containing solution. After incubation and washing to remove impurities, exosomes displaying CD63 expression can be isolated. (**C**) Because the aptamer used in this system undergoes spatial changes after 0.5 M NaCl incubation (mild condition) and releases the conjugate, the natural structure and biological functions of the collected exosomes are ensured.

CD63 is one of the mostly described generic biomarkers of exosomes. Over the past decades, various types of anti-CD63 antibodies have been exploited for the development of immunoaffinity-mediated exosome separation. Even though ensuring highly purified exosome separation, the non-physiological condition elution buffer (for exosome/antibody separation) used in immunoaffinity-based exosome isolation could irreversibly compromise the biological functions of the isolated exosomes. However, for many of the downstream applications such as exosome-mediated therapeutic trials, obtaining native state exosomes is imperative. In this work, via developing aptamer sequences using 0.5 M NaCl as elution buffer at the late stage of selection, aptamers with binding affinity that are easily modulated under such mild conditions have been developed. As shown in Figure 6, streptavidin beads were firstly incubated with biotinylated aptamers to form the aptamer/bead complex. After incubating with exosome containing solution, the surface CD63 protein and therefore the whole exosome could be specifically isolated by the bead-conjugated CD63-1 aptamer. As the tertiary structure of CD63-1 sequence could be altered via 0.5 M NaCl and release its target, this provides a simple method for native exosome isolation.

**Figure 7:**
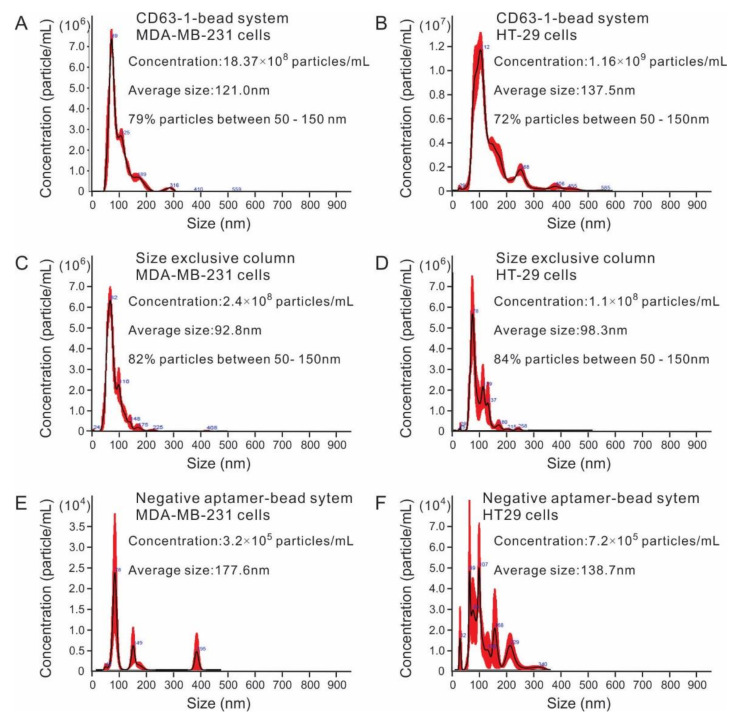
Analysis of exosomes collected by different methods. (**A**,**B**) NTA analysis of MDA-MB-231 and HT29 exosomes isolated by bead/aptamer system; (**C**,**D**) NTA analysis of MDA-MB-231 and HT29 exosomes isolated by size exclusion chromatograph method; (**E**,**F**) NTA analysis of MDA-MB-231 and HT29 exosomes isolated by a negative aptamer (CD63-6)-based bead system.

As shown in Figure 7A,B, after incubating 10 mL conditioned cell culture medium with CD63-1/bead system for 1h at room temperature, followed thorough washing and a final step 10 min 1 mL NaCl (0.5 M) incubation, up to 8.37 × 10^8^/mL and 1.16 × 10^9^/mL exosome vesicles (Supplementary Figure S3) were collected from MDA-MB-231 and HT29 cell culture, with 79% and 72% particles having particle size between 50–150 nm for MDA-MB-231 and HT29 cells, respectively, typical for the exosomes observed elsewhere [24]. As a positive control, the commonly used commercial size exclusion chromatography exosome isolation column was employed. After adding an equal amount of cell culture medium (to fit the SEC column, the 10 mL medium was firstly concentrated to 0.5 mL) with the aptamer/bead method, the exosome samples were collected in 2.5 mL PBS solution prior to NTA analysis. As shown in Figure 7C,D, the exosome amount for MDA-MB-231 and HT29 cells were 2.4 × 10^8^/mL and 1.1 × 10^7^/mL, respectively. Given the concentration factor of both methods (0.5 mL elute for bead/aptamer method and 2.5 mL elute for the SEC method), the exosome yield would be comparable for these two methods. As a negative control, we also prepared a negative bead system using the non-binding CD63-6 sequence. As shown in Figure 7E,F, the collected particles most likely resulted from the nonspecific weak binding between particles and beads, with the yield being more than 100-fold lower than both CD63-1 bead system and the SEC-based isolation. In recent years, SEC method is gaining increasing popularity for its simplicity and lack of damage to exosome structure and functions. The innate state of exosomes could be equally kept in exosomes prepared by the tested bead/aptamer system. This is because with the developed bead/aptamer system, the aptamer/exosome separation was triggered by a relatively mild condition of 0.5 M NaCl incubation. Since NaCl concentration from 0.5 to 1 M has been commonly used for protein purification [42], the native state of exosomes could be ensured. Furthermore, the buffer system of the collected exosomes via the bead/aptamer system could be easily replaced through a regular dialysis step.

Notably, in both MDA-MB-231 and HT29 cell lines, exosomes derived from the aptamer/bead system showed larger particle size compared with SEC (Figure 7). This is most likely because of the volume segments selected in the SEC method. As known, in the SEC method, the particles travel through porous materials and cause particles to run through the column according to their size, with the large particles eluted earlier than the smaller particle. The relatively smaller size of the exosome collected in the SEC method may due to the collection window (volume fragment, from 3–5.5 mL in this case for qEV/original SEC (Izon Science)). Due to the collected exosomes only representing a fraction of the total exosome, exosomes prepared by this method may result in the loss of information during downstream analysis and applications. The aptamer based immunoaffinity method represents an obvious advantage in this aspect, because it allows isolating exosomes (CD63 positive) irrespective of their size. In addition, as demonstrated in our study, with similar yield, the bead/aptamer method is more simplified. Differing from the aptamer/bead system, which can directly processing the original samples and easily adjust the volume of the collected exosomes for downsteam applications, the SEC method needs to pre-concentrate samples (to 0.5 mL in this case) to fit the SEC column. In addition, the exosomes prepared by SEC can only be collected to a fixed volume (2.5 mL in this case); additional volume adjustment and desalting steps are necessary in most cases.

In summary, via a carefully designed competitive-SELEX, companied by NaCl elution, we have developed novel CD63 protein targeting aptamer sequences, displaying not only specific binding capacity to native CD63 protein, but also, through a simple incubation with 0.5 M NaCl, the capacity to alter the tertiary structure of the aptamer sequences and release the bound CD63 protein molecules. Further investigation disclosed that the selected CD63-1 aptamer could effectively recognize cell surface CD63 protein and hold potential to be used as a substitute in clinical immunohistochemistry assay for cancer diagnosis. Importantly, after developing a magnetic bead-based exosome immuno-separation system using CD63-1 aptamer, it was found that this bead-based system could effectively isolate exosomes from MDA-MB-231 cell culture medium. Importantly, the isolated native exosomes could be efficiently eluted via 0.5 M NaCl incubation, providing a novel strategy for isolating exosomes from clinical samples for various extracellular-vesicle-based basic and applied investigations.

## 3. Materials and Methods

### 3.1. Cell Lines and Cell Culture

MDA-MB-231, HT29, and HEK293T cells were bought from the American Type Culture Collection. The three cell lines were all cultured in DMEM (Invitrogen, 12800-017), supplemented with 10% FBS (Hyclone, A50111) and 1× Glutamax (Life Technologies, 35050-061) at 5% CO_2_ and 37 °C. The CD63 overexpressed HEK293T cell control was established by transient transfection of a pEX-3 expression vector driven by a CMV promoter with a CD63 cDNA. The transfection was conducted via Lipofectamine 2000, according to the manufacturer’s instruction.

### 3.2. Buffers and Sequence Information

Binding buffer: pH 7.4 PBS containing 2.0 mM MgCl_2_, 0.02% Tween 20, and 100 µg/mL Salmon Sperm DNA (ThermoFisher, 15632011); wash buffer: pH 7.4 PBS containing 2.0 mM MgCl_2_ and 0.02% Tween 20; ssDNA library: 5′-TAGGGAAGAGAAGGACATATGAT N43 TTGACTAGTACATGACCACTTGA-3′(N represents 30% C, 30% G, 20% A, 20% T); forward primer: 5′ TAGGGAAGAGAAGGACATATGAT3′; reverse primer: 5′TTTTTTTTTTTTTTTTTTTT/iSp9/TCAAGTGGTCATGTACTAGTCAA3′. The initial ssDNA library sequence was synthesized by Integrated DNA Technologies (IDT), with a central 43 nucleotide randomized sequence and primer binding sites on both ends. To facilitate the binding assessment, 5′ biotinylated forward primer was used to introduce 5′biotin to the resulted sub-libraries. The reverse primer was conjugated with 20 consecutive dT nucleotides via a triethylene glycol spacer to facilitate denaturing Urea-PAGE gel-based single stranded DNA separation. CD63-1 aptamer: 5′ TAACACGACAGACGTTCGGAGGTCGAACCCTGACAGCGTGGGC3′; CD63-2 aptamer: 5′ TAACCACCCCACCTCGCTCCCGTGACACTAATGCTAATTCCAA 3′.

### 3.3. CD63 Protein Target Immobilization

Human recombinant CD63 protein was expressed in mammalian cells with a sequence (Ala 103-Val 203) of human CD63 fused with a poly-histidine tag at the C-terminus (Sino Biological, Cat. 11271-H08H, Beijing, China). 100 pmol His-tagged CD63 protein was dissolved in 500 µL of binding buffer and immobilized onto Ni-NTA agarose beads (Thermofisher, R90101, Waltham, MA, USA) via 1-h room-temperature incubation for aptamer identification. As a negative selection control, an equal amount of an irrelevant protein, His-tagged CD81, with a comparable molecular weight with CD63 was immobilized on Ni-NTA agarose beads via an identical method as CD63 protein.

### 3.4. SELEX Selection

Single-stranded DNA (ssDNA) library pool (4 nmol for initial selection, 100 pmol for each round from round 2) was dissolved in 500 µL of binding buffer and denatured at 95 °C for 10 min, followed by placing it on ice for 10 min. Then, the ssDNA library was loaded to the plate and incubated with CD63 for 1 h at room temperature. To make sure the selected aptamers can bind to the native state CD63 protein and eliminate the non-specific binding of oligonucleotides to agarose beads, after incubation and extensive washes, 0.5 nmol native state CD63 (around 50 times of the immobilized protein bait, the binding capacity of the well is 9 pmol/well) was loaded to the immobilized CD63/ssDNA mix for ssDNA elution. The eluted ssDNA sequences were enriched by Emulsion PCR, and ssDNAs were prepared from the PCR product by conducting denaturing PAGE gel-based ssDNA separation for the next round of SELEX. Negative selection was introduced from round one using CD81, an irrelevant protein. Simply, before incubating ssDNA libraries with CD63 target, the ssDNA libraries were incubated with immobilized CD81 protein for 1 h, followed by transferring the supernatant to bead immobilized CD63 protein. The stringency of the selection was improved to facilitate the identification of high-affinity aptamers by adjusting aptamer concentrations (1 nmol for the initial round, 100 pmol afterwards), incubation time (1 h for round 1–4 and 0.5 h afterwards), the time of washes (from three washes for round 1 gradually increased to six washes for round 10), and the CD63 amount (100 pmol for round 1–3, 50 pmol for round 4–7, 20 pmol afterwards). At the last two rounds, after positive selection and thorough washing, 200 µL of 0.5 M NaCl was added to the aptamer/bead system to dissociate sequences from CD63 protein under 0.5 M NaCl. The enrichment of aptamer sequences was examined via an ELISA assay (detailed in Section 3.6). The SELEX was terminated once no further improvement in binding was recorded between sub-library pools from successive three rounds. A detailed procedure about this experiment was demonstrated in the Table S1. Enriched aptamer candidates were then processed for the next-generation sequencing using a MiSeq platform to select potential aptamer binders.

### 3.5. Emulsion PCR

To avoid the PCR bias caused by traditional PCR, an improved emulsion PCR was performed in this work. The oil phase and water phase were prepared according to the Table 1. After preparing the indicated oil phase and water phases, the oil phase and water phase were mixed by ratio of 3:1. The mixture was then subjected to a 2 min shaking at 30 Hz using a tissue lyser (Qiagen, TissuelLyser II, Canton, MA, USA). The PCR program was as follows: initial 95 °C for 1 min, followed by 30 cycles of 95 °C 30 s, 56 °C 60 s, and 72 °C 60 s.

### 3.6. ELISA Assay

Similar to a previous report [16], firstly 1 µg of CD63 protein was added to 200 µL PBS and loaded to the Ni-NTA coated plate (Thermofisher, Cat. 15442) at RT for 1 h. After that, blocked wells with 100 µg/mL of salmon sperm DNA (ThermoFisher, 15632011) were loaded for 1 h at RT. After washing three times with PBS, wells were incubated with 200 µL of different concentration of biotinylated aptamers at RT for 0.5 h. Then, the wells were washed three times with wash buffer, followed by adding HRP-conjugated anti-biotin antibody (Abcam, ab19221, Cambridge, MA, USA) for 1 h. After thorough washing, the fluorescence signal was analysed by a plate reader using QuantaBlu Fluorogenic Peroxidase Substrate Kit (Thermofisher, 15169), according to the manufacturer’s manual.

### 3.7. FACS Assay

Firstly, cells were blocked on ice using a blocking buffer (PBS plus 100 µg/mL salmon sperm DNA and 1% BSA). After that, cells were washed twice using a binding buffer, followed by incubation with various concentrations of aptamers (FAM-labelled, prepared using binding buffer) for 1 h at RT. After the incubation, cells were washed three times with binding buffer. After resuspending in in 200 µL binding buffer, flow cytometry assay was performed (BD FACSVia™ System, USA). The mean fluorescence intensity (MFI) of the aptamer treated cells was subtracted by that of the unstained cells to generate the MFI of specific binding.

### 3.8. Fluorescence Microscopy Imaging

The aptamer preparation steps were the same manner with the FACS assay (Section 3.7). After removing the supernatant, cells were treated with blocking buffer for 1 h at RT, followed by washing twice. The cells were then incubated using 200 nM aptamers for 30 min at RT. At the last 15 min of the incubation, nucleic stain was performed using Hoechst 33,342 (3 µg/mL; Sigma, B2261). The cells were then thoroughly washed and visualized using a FV3000 Confocal Laser Scanning Microscope (Olympus, Tokyo, Japan).

### 3.9. Immunohistochemistry Assay

The breast cancer sections were firstly fixed by acetone (Sigma-Aldrich, V800023) for 30 s at RT. Then the sections were blocked in DMEM medium plus 0.1 mg/mL tRNA and 10% goat serum for 1 h at RT. After the blocking step, 200 μL of 400 nM Biotin labelled aptamer (or 1:1000 diluted anti-CD63 antibody) were added to the slide and held for 30 min. After the incubation, sections were washed three times using serum-free DMEM medium in a coplin jar, 5 mL per wash. The sections were then incubated with HRP-conjugated anti-Biotin antibody (Abcam, ab19221, Cambridge, MA, USA) at a concentration of 1:300 for 1 h at RT, followed by DAB (Vector, SK-4100) development for 5 min and thorough washing. Next, nucleus stain was performed via Harris hematoxylin solution (Vector, H-3502, Burlingame, CA, USA) at RT for 5 min. The differentiation was then performed using 1% acid alcohol for 5 s, followed by 1 min bluing in Scott’s tap water substitute (Sigma, S5134, Saint Louis, MO, USA). The dehydration was then conducted in 70%, 95% and absolute alcohol, 2 min each. Lastly, the sections were mounted using histolene (Sigma, H2779, Saint Louis, MO, USA) and forwarded for imaging.

Breast cancer samples used in this project were obtained through the First Affiliated Hospital of Dalian Medical University (Dalian, P.R. China) from consenting patients. Application of clinical cancer biopsies and associated procedures were approved by the ethics committee of the Dalian Medical University [Number: SCXK(Liao) 2017-0129].

### 3.10. Construction of CD63 Aptamer-Based Exosome Immunoaffinity Bead System

Biotinylated CD63 aptamer of 5 μM (500 μL) was heated at 95 °C for 10 min and chilled on ice for 5 min to reconstruct its conformational structure. At the same time, 50 μL of streptavidin beads (Thermofisher, 11205D) was washed using PBS buffer, followed by resuspending in 500 μL PBS, including 5 μM CD63 aptamers. After 1 h incubation, the bead/aptamer mixture was washed thoroughly using PBS.

Cells were incubated in 5% CO_2_ at 37 °C and were grown in T-75 flasks until they reached 80–90% confluency, then washed three times with PBS and incubated in the 10 mL PBS plus 0.5% exosome-depleted FBS for 24 h. After removing cells, apoptotic bodies and cell debris via centrifugation at 2000× *g* for 20 min at 4 °C, adding the supernatant to bead/aptamer mixture for 0.5 h incubation on a rotator at room temperature. After the incubation, the beads were washed using PBS plus 2 mM MgCl_2_ and 0.02% Tween 20, followed by a 10 min incubation in 500uL of 1 M NaCl. The eluted exosomes were then collected and analyzed.

### 3.11. Preparation of Exosomes Using Size Exclusive Chromatography (SEC)

Cells were incubated at 37 °C in 5% CO_2_ and were cultured in T-75 flasks until they reached around 80–90% confluency. Cells were then washed three times with PBS and incubated in the exosome-harvesting medium for 48 h. Exosome-harvesting medium includes DMEM plus 0.5% exosome-depleted FBS, as described previously [24]. The conditioned medium (CM) was harvested and centrifuged at 2000× *g* for 20 min at 4 °C to remove cells, apoptotic bodies, and cell debris. The medium was then concentrated around 10 times via Centricon^®^ Plus-70 centrifugal filter with a 10 K molecular weight limit (Millipore, MA, USA) under 3500× *g* for 20 min. An invert spin was then performed at 1000× *g* for 2 min at 4 °C. The concentrated media was added to a qEV/original SEC (Izon Science); the fragment between 3–5.5 mL was collected and analyzed.

### 3.12. Nanoparticle Tracking Analysis (NTA)

Exosome-containing samples were assessed via NTA using Nanosight model NS300 equipped with 405 nm blue laser and a CMOS camera. The samples were diluted 100 times in PBS, and videos were recorded using camera level 12 under controlled fluid flow mode with a pump speed set to 40 μL/min. The results were then assessed via Dev Build 3.2.16 with the optimised detection threshold. The camera gain was set at 146 with a variable shutter length at 30 frames per second. The data was collected through the area under the histogram for average of triplicate experiments.

### 3.13. Statistical Analysis

The data were analysed using GraphPad Prism 3.03. An unpaired *t* test was performed for comparisons between two different groups. Except where specifically noted, all results were average of triplicates, and values were prepared as means ± SEM. A *p* value of less than 0.05 was considered statistically significant.

## Figures and Tables

**Table 1:** The recipe of the emulsion PCR.

Oil Phase (µL)	
DC5225C	500 µL
DC193	500 µL
CD556	1200 µL
Span 80	80.5 µL
Tween 80	80.5 µL
Tween 20	22.58 µL
Total volume	2383.58 µL
Water phase (PCR master mix, µL)	
5× PCR buffer	100 µL
dNTP (10 mM)	10 µL
Primer mix (20uM)	25 µL
Polymerase	10 µL
Template	25 µL
BSA (5%)	5 µL
Water	320 µL
Total volume	500 µL

## Supplementary Materials

The following are available online, Figure S1. Evaluation of the relative binding capacity of 8 aptamer candidates to CD63 protein, Figure S2. NaCl incubation reduces the binding of CD63-1 and CD63-2 aptamers to CD63 protein. Figure S3. Screen capture of the NTA video showing the isolated exosomes by SEC and aptamer/bead methods. Table S1: Eight selected aptamers candidates for affinity assay.

## Abbreviations

NTA	Nanoparticle Tracking Analysis
SELEX	Systematic evolution of ligands by exponential enrichment
SEC	Size-exclusion chromatography
