# Supplementary material for: Development of a CD63 Aptamer for Efficient Cancer Immunochemistry and Immunoaffinity-Based Exosome Isolation
Source: Molecules. 2020 Nov 27;25(23):5585. doi: 10.3390/molecules25235585 (PMC7730289; doi:10.3390/molecules25235585)
Supplement: Supplementary file 1 [file molecules-25-05585-s001.pdf]

molecules

Article

# Development of a CD63 Aptamer for Efficient Cancer Immunochemistry and Immunoaffinity-Based Exosome Isolation

**Zhenguo Song [1,†], Jun Mao [2,†], Roberto A. Barrero [3], Peng Wang [4], Fengqiu Zhang [5,*] and Tao Wang [4,*]**

[1] Department of Pharmacy, Affiliated Cancer Hospital of Zhengzhou University, Zhengzhou 450008, China; zlyysongzhenguo1677@zzu.edu.cn
[2] College of Basic Medical Sciences, Dalian Medical University, Dalian 116044, China; maojun1116@163.com
[3] eResearch Office, Division of Research and Innovation, Queensland University of Technology, Brisbane City QLD 4001, Australia; roberto.barrero@qut.edu.au
[4] College of Nursing and Health, Zhengzhou University, Zhengzhou 450001, China; pengwang@zzu.edu.cn
[5] Henan Key Laboratory of Ion-beam Bioengineering, Zhengzhou University, Zhengzhou 450000, China
* Correspondence: zhangfengqiu@zzu.edu.cn (F.Z.), wangtaomary@zzu.edu.cn (T.W.); Tel: +861393839312 (F.Z.); +61432684878 (T.W.)
† These authors contributed equally to this work.

**Supplementary Table 1.** Sequence information of the top 8 aptamer candidates.

| No. | Sequence (without primer binding site) | Frequency |
|---|---|---|
| CD63-1 | TAACACGACAGACGTTCGGAGGTCGAACCCTGACAGCGTGGG | 8.37% |
| CD63-2 | TAACCACCCCACCTCGCTCCCGTGACACTAATGCTAATTCCAA | 9.64% |
| CD63-3 | CGACATGCCTGTGCTAGCCGAACCATGCTGCAATTCACTCGTG | 4.17% |
| CD63-4 | AACACGACCCTTGTTCGCGAGGCACAGTCCATGCTGGTCGTGA | 3.87% |
| CD63-5 | GGCTACGACGTACCTGTGAAAGCCGAACATATTGCGAGTGGGC | 1.99% |
| CD63-6 | TACCTACGACATGCCTGTGCTAGCCGAACCATGCTGAGGTCGT | 1.87% |
| CD63-7 | ACCCAGACTAGGGTTCGATCATCACAGAACGAGGGCGTCTCCG | 1.42% |
| CD63-8 | CGACCGGCAAAGTTCGTCCACCGAACAGATCGGCTGTCGTGCC | 0.97% |

Note: the sequences meeting the criterial (89nt total length with the eprimer binding sites) was 242,342 in total.

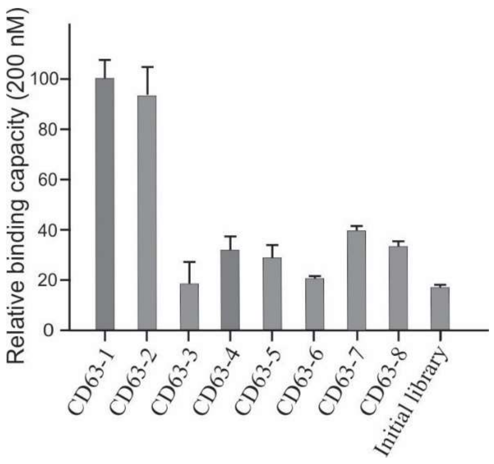


**Supplementary Figure 1.** Evaluation of the relative binding capacity of eight aptamer candidates to CD63 protein. The binding capacity was assessed by ELISA assay. After a one-hour blocking step using salmon sperm DNA (10μg/100μL/test), 200 nM of the indicated aptamer candidates (biotinylated) were incubated with the immobilized CD63 protein for 0.5 h, followed by HRP conjugated anti-biotin antibody incubation. The fluorescence intensity was measured by a plate reader (using the QuantaBlu Fluorogenic Peroxidase Substrate).

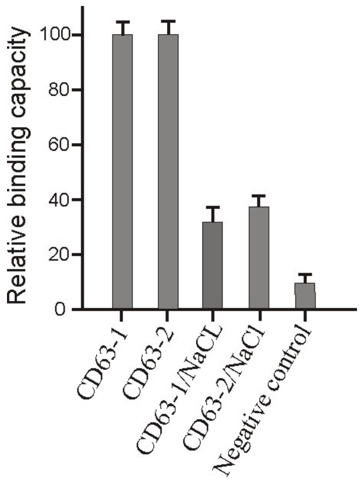


**Supplementary Figure 2.** NaCl incubation reduces the binding of CD63-1 and CD63-2 aptamers to CD63 protein. Biotinylated CD63-1 and CD63-2 aptamers (two groups for each aptamer) were incubated with CD63 protein in a 96-well nickel plate for 30 min at a concentration of 200 nM. After the 30 min incubation, one group of each aptamer were further incubated with NaCl (0.5 mM) for 10 min. After thorough washing and incubating with anti-biotin antibody for 1 h, the fluorescence intensity was measured by a plate reader (using the QuantaBlu Fluorogenic Peroxidase Substrate). The binding of each group was calculated after subtracting the mean fluorescent intensity of the binding of the protein only group. The original library (biotinylated) served as control *, P ≤ 0.05.

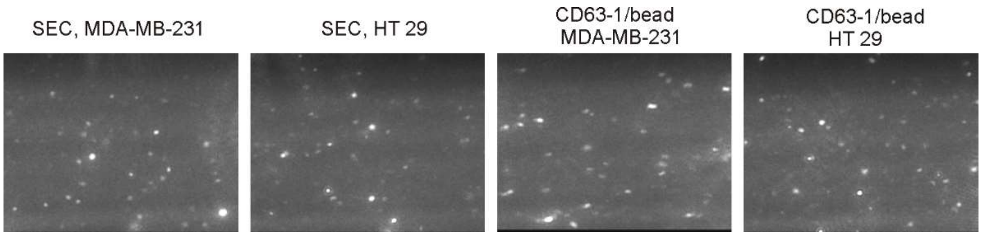


**Supplementary Figure 3.** Screen capture of the NTA video showing the isolated exosomes by SEC and aptamer/bead methods.
